# Sample Size Analysis for Machine Learning Clinical Validation Studies

**DOI:** 10.3390/biomedicines11030685

**Published:** 2023-02-23

**Authors:** Daniel M. Goldenholz, Haoqi Sun, Wolfgang Ganglberger, M. Brandon Westover

**Affiliations:** 1 Department of Neurology, Beth Israel Deaconess Medical Center, Boston, MA 02215, USA; 2 Department of Neurology, Harvard Medical School, Boston, MA 02215, USA; 3 Department of Neurology, Massachusetts General Hospital, Boston, MA 02114, USA

**Keywords:** statistics, machine learning, power calculation

## Abstract

Background: Before integrating new machine learning (ML) into clinical practice, algorithms must undergo validation. Validation studies require sample size estimates. Unlike hypothesis testing studies seeking a *p*-value, the goal of validating predictive models is obtaining estimates of model performance. There is no standard tool for determining sample size estimates for clinical validation studies for machine learning models. Methods: Our open-source method, Sample Size Analysis for Machine Learning (SSAML) was described and was tested in three previously published models: brain age to predict mortality (Cox Proportional Hazard), COVID hospitalization risk prediction (ordinal regression), and seizure risk forecasting (deep learning). Results: Minimum sample sizes were obtained in each dataset using standardized criteria. Discussion: SSAML provides a formal expectation of precision and accuracy at a desired confidence level. SSAML is open-source and agnostic to data type and ML model. It can be used for clinical validation studies of ML models.

## 1. Introduction

Reports of opportunities for machine learning (ML) to improve clinical care are being published at an accelerating rate [1]. Proof-of-concept studies with a clinical ML model are now common. However, before adopting such models into clinical practice, they should undergo *validation* [2]. Clinical validation provides a confirmation that the proposed algorithm can be generalized to situations or patients not previously encountered and helps to clarify the limitations of an algorithm [3].

Clinical validation studies are often expensive, time consuming, and may expose subjects to risk. Therefore, researchers need to determine the minimum number of samples/events/patients needed to verify an algorithm with a specified confidence level. Most clinical investigators are familiar with sample size calculations designed to test a yes/no question, couched in terms of the familiar machinery of significance testing, i.e., the number of subjects or events needed to provide a given level of power to reject a null hypothesis with a specified level of type I errors. By contrast, the goal of a validation study for a predictive model is to estimate model *performance measures.* That is, the role of a clinical validation study is to measure model performance—accurately and precisely. Accuracy means low bias (small distance from “true” estimates). Precision means high certainty (small confidence intervals). Thus, the goal of a clinical validation is conceptually distinct from hypothesis testing [4]. 

In addition, because ML models for medical problems can be challenging to interpret (e.g., deep learning with millions or billions of parameters), and increasingly deal with data generated by processes not well described by conventional statistical models, sample size calculations are not always straightforward for predictive models developed using ML techniques. 

Here, we propose a general algorithmic approach for Sample Size cAlculation for ML clinical validation studies (SSAML). Unlike several other proposed techniques [5,6,7], SSAML makes no assumptions about the specific ML technique being validated, or about the type of data involved. By being model agnostic, SSAML permits classical statistical predictive models (such as regression models) or modern machine learning algorithms to be tested in the same way. This approach builds on earlier work by Collins et al. [4]. We provide open-source computer code that implements SSAML, so that other investigators in translational medicine can use the technique to estimate the sample size needed for their own ML model validation studies. 

## 2. Materials and Methods

The data were obtained in a de-identified format from three previously published studies on neurologic prediction models. The first was a study of the Brain Age Index (BAI) [8], which used sleep EEGs from participants in the Sleep Heart Health Study [9]. The BAI study estimated the “brain age” based on a transformed linear regression model of the EEGs which was then compared to the participant’s actual age. Using survival analysis in combination with BAI, it was found that life expectancy could be estimated [10]. The second study (COVA) evaluated the risk of hospitalization, ICU admission, and death from COVID-19, utilizing an ordinal regression model [11]. The third study from Seizure Tracker™ (ST) developed a seizure forecasting system based on de-identified electronic seizure diaries using an artificial neural network model (deep learning) [12]. The ST study was exempted by the Beth Israel Deaconess Medical Center Institutional Review Board, 2017D000488. The COVA and BAI studies were exempted by the Mass General Brigham Institutional Review Board, 2013P001024. The basic characteristics of each study are summarized in Table 1.

Our technique, SSAML consist of five steps:

- **STEP (1)** Specify performance metrics, including measures of model *discrimination* (ability to distinguish cases from controls) and *calibration* (how well the model’s risk predictions match observed case rates). For discrimination, we used the area under the receiver operating curve (*AUC*) for binary classification tasks (ST and COVA); we used the Harrell’s *C-index* (a generalization of *AUC*) for our survival model task (BAI). For calibration, we employed calibration *slope* and calibration-in-the-large (*CIL*) [13].
- **STEP (2)** Specify the required precision (relative width of confidence intervals, or *RWD*) and accuracy (percent bias or *BIAS*). We used cut-offs of ≤0.5 for precision and ±5% for accuracy. Let *CI* be the difference of the limits of the confidence interval, and *trueValue* be the estimate of the true value being estimated from the population. Then, *RWD* is given by:
  (1) $$RWD=\frac{CI}{trueValue}$$
  For a given trial attempting to approximate the *trueValue*, let the approximation be called *estimate*. The accuracy or *BIAS* is then given by:
  (2) $$BIAS=\frac{estimate-trueValue}{estimate}$$
- **STEP (3)** Specify the required confidence (probability that the *CI* includes the true value, i.e., “coverage probability” or *COVP*). We recommend 95%. *COVP* is given by:
  (3) $$COVP=Prob\left[trueValue\;\in CI\right]$$
- **STEP (4)** For increasing sample sizes, calculate the expected precision (*RWD*) and accuracy (*BIAS*) that is achievable, subject to the coverage probability requirement (*COVP* > 95%). This should be calculated for each metric (*slope*, *AUC* (or *C-index*), and *CIL*).
- **STEP (5)** Choose the minimum sample size that meets the requirements. Thus, at the minimal sample size, all three metrics (*slope*, *AUC/C-index*, *CIL*) must satisfy these equations:
  (4) RWD<0.5
  (5) $$\left|BIAS\right|<5\%$$
  (6) COVP>95%
  Let *N* represent a given number of samples. The sample size required for each metric (*slope*, *AUC/C-index*, *CIL*) to satisfy Equations (4)–(6) is:
  (7) $$S_{slope}=\underset{RWD<0.5,\;\left|BIAS\right|<5\%,COVP>95\%}{\min}N$$
  (8) $$S_{AUC\;}=\underset{RWD<0.5,\;\left|BIAS\right|<5\%,COVP>95\%}{\min}N$$
  (9) $$S_{CIL}=\underset{RWD<0.5,\;\left|BIAS\right|<5\%,COVP>95\%}{\min}N$$
  Taking the maximum sample size of the three metrics ensures that all metrics will meet all criteria. Therefore, *S_overall_* represents the ideal sample size from SSAML:
  (10) $$S_{overall}=\underset{i=slope,AUC,CIL}{\max}S_{i}$$

The ML approach to be validated is run on a bootstrapped sample of the data, and the chosen discrimination and calibration metrics are computed; in our examples we used calibration *slope*, *AUC* or *C-index*, and *CIL*. These calculations can be performed on real data or on simulated data that captures the investigator’s hypothesis about the data generating process. This calculation is repeated N times from randomly chosen samples with replacement (bootstrapping), allowing the calculation of mean and confidence intervals from the performance metrics. Bootstrapping is repeated M times (i.e., double bootstrapping [14]) to obtain mean estimates of *RWD* and *BIAS*, and for *COVP*. If *COVP* < 95% in any metric, the confidence interval (CI) is enlarged, and the double bootstrapping procedure is repeated. If all metrics meet criteria, then this process is repeated with a larger number of patients (or events). The smallest number of patients or events that satisfies all selection criteria is then chosen.

For each of our three illustrative datasets, we ran SSAML for a set of four possible sample sizes. The COVA dataset has a binary outcome measured once per patient, estimated using an ordinal regression. The BAI dataset has a numerical outcome measured once per patient with censorship (i.e., survival analysis), estimated using a Cox proportional hazard model. For BAI, we estimated the number of events needed for a validation study, rather than the number of patients needed. The ST dataset has a binary outcome which was measured in a timeseries and forecasted repeatedly in each patient, estimated with deep learning. It is noted that the choice to use events rather than patients is at the discretion of the user—either way is essentially equivalent, but we opted to illustrate both. 

In addition, we generated simulated datasets to explicitly investigate the effect of different numbers of model input features (10 and 100), class imbalance ratios (1:1 and 1:10), noise levels (input label were swapped at a rate of 10% and 20%) on the confidence intervals of the metrics from SSAML. These simulated datasets were fit with a logistic regression model, and SSAML was run on each. Simulations of different levels of skew and outliers were also performed using a subset of the above datasets. The purpose of these simulations was to show that numbers of inputs, imbalance ratios, and noise levels could impact SSAML in expected ways.

Open-source code for SSAML and the simulations are available (https://github.com/GoldenholzLab/SSAML).

## 3. Results

The main features of each dataset are summarized in Table 1. Each dataset presents a unique challenge in terms of type of data, machine learning algorithm, and unique features. These examples were chosen to show the variety of applications that SSAML can manage successfully. 

The results from SSAML are summarized in Table 2. The results from the smallest confidence interval for each patient or event size that met the *COVP* ≥ 95% condition is shown. Using this data table, it is possible to estimate a minimum sample size required for a clinical validation study that all meets the criteria established here (*RWD* < 0.5, *BIAS* < 5%, *COVP* > 95%). These numbers are as follows: BAI, 1500 patients; COVA, 150 events; and ST, 40 patients.

**Figure 1:**
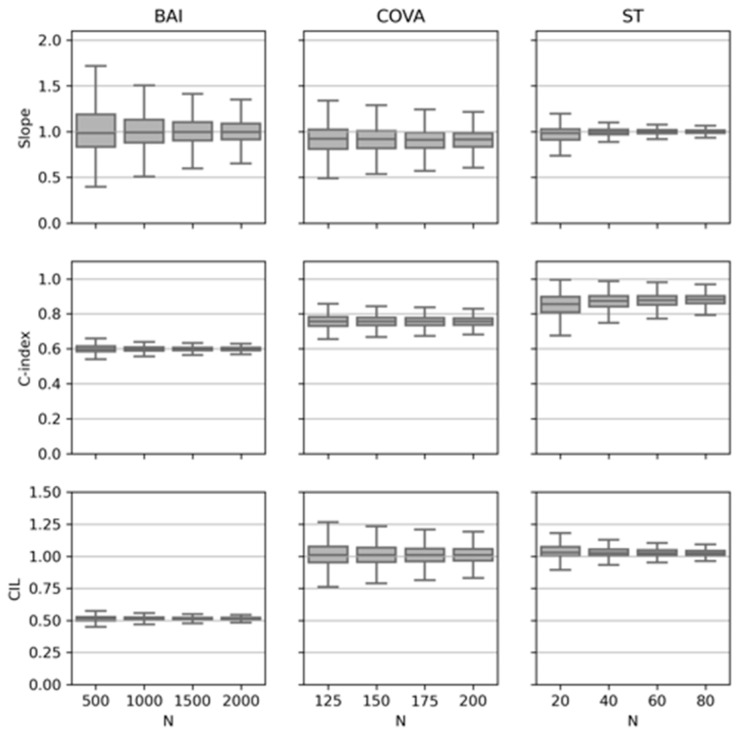
Narrowing confidence regions with increased number of patients/events. Shown here are three example datasets, one per column: Brain Age Index (BAI) [8,10], COVID-19 risk Assessment (COVA) [11], and Seizure Tracker™ (ST) [12]. Each row indicates metrics for model performance: calibration slope (*slope*), area under the receiver operator curve or Harrell’s c-index (*C-index*), and calibration-in-the-large (*CIL*). In each subplot, as the number of patients or events (*N*) increased, the confidence interval narrowed. When the desired performance level was reached, this represents the minimum powered study.

The three metrics for calibration and discrimination are plotted in Figure 1 for each of the three datasets. This plot illustrates the point that using increasingly larger sample sizes reduces the uncertainty (narrows the confidence intervals) for estimates of each of the metrics. In all three cases, the confidence intervals around the *slope* narrowed with increasing numbers of patients/events converging close to 1.0 as expected. The *C-index* converged to different final values depending on the dataset. BAI tended towards 0.6, COVA to just under 0.8, and ST to just under 0.9. These differences reflect the underlying discrimination capabilities of the specific models. The *CIL* values for BAI were considerably lower than ST and COVA. This, combined with the *slope*, indicated better calibration for ST and COVA compared with BAI. Regardless of the differences in how accurate or precise these models may be, SSAML clarifies how many samples are needed for a validation study in each case.

**Figure 2:**
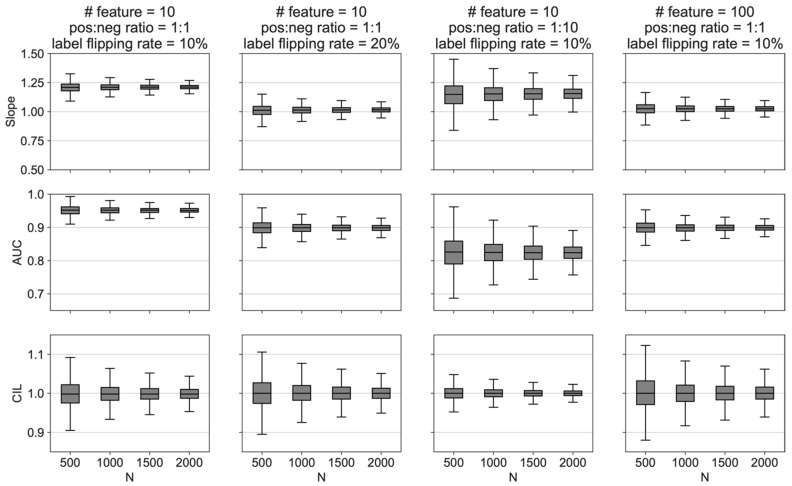
We generated four simulated datasets (in four columns) representing a binary classification problem that have different numbers (#) of features (10 and 100), positive vs. negative class imbalance ratios (1:1 and 1:10), and noise levels (label flipping rate of 10% and 20%). Each dataset was fit using a logistic regression. The fitted data were then fed into the SSAML algorithm. The purpose is to explore the behavior of the confidence intervals of the calibration *slope* (top row), *AUC* (middle row), and *CIL* (bottom row) under known conditions. Several simple observations can be made here: (1) the confidence intervals decreased with increasing *N* in all conditions; (2) the calibration *slope* was sensitive to different conditions; (3) increased noisy or class imbalance decreased the *AUC*; (4) *CIL* was not sensitive to the different conditions tested; and (5) evaluating all three metrics (*slope*, *AUC*, and *CIL*) was important.

Figure 2 summarizes simulations using two different numbers of features (10 vs. 100), two class imbalance ratios (1:1 vs. 1:10), and two noise levels (label flipping rate of 10% vs. 20%). First, the confidence intervals decreased with increasing *N* in all conditions. Next, the calibration slope was sensitive to the different conditions (features, imbalance, and noise). Additionally, increased noise or imbalance independently decreased the *AUC*. Additionally, *CIL* was not sensitive to features, imbalance, or noise. Finally, it is helpful to review the *slope*, *AUC*, and *CIL* to get a global sense of how well a given number of patients fits the model. Overall, the behavior of the simulated data helped to confirm intuitions about how SSAML would behave under various conditions.

Next, we tested how the SSAML is affected by outliers. We used the simulated dataset with 100 features, balanced class ratio (to not introduce irrelevant factor), and label flipping rate of 10% (a near perfect case to not introduce irrelevant factors). We varied the percentage of outliers to 0%, 5%, and 10% by randomly multiplying the specified proportion of the input features by 10. We ran SSAML on *N* = 500, 1000, 1500, 2000, and 2500. The result indicated that when there is no outlier, we need *N* = 1000; when the outlier percentage was 5%, we need *N* = 1000 (no change due to the coarse level of Ns tested); when the outlier percentage was 10%, we need *N* = 1500. When we compared the *RWD*, *BIAS*, and *COVP* metrics, with an increasing percentage of outliers, all *RWD* metrics increased with no change in all *BIAS* and *COVP* metrics. The increasing *RWD* metrics confirmed the conclusion that more samples are needed when there are more outliers.

We also tested how SSAML is affected by skewed data distribution. We used the same simulated dataset as in the outlier test above. To achieve skewed distribution, we first sorted the dataset according to the feature with median *t*-test *p*-value with class labels, which represents an average feature; we then took the subset of the dataset that maximizes the skewness of that feature. In this way, we increased the skewness while preserving the mapping between features and class labels. The normality test had a *p*-value < 0.0001 which rejects the null hypothesis of being a normal distribution at alpha = 0.05 level. We ran SSAML on *N* = 500, 1000, 1500, 2000, and 2500. The result indicated that when there was no impact of a skewed distribution on any *RWD*, *BIAS*, or *COVP* metrics, and hence no impact on the sample number needed. The reason is that, in realistic settings, although the data is skewed, the mapping between data and class labels is not affected, hence not affecting the prediction performance. This is different to having outliers which affects the prediction performance.

## 4. Discussion

As demonstrated here, SSAML provides algorithmic sample size calculations for validation of predictive models involving machine learning in clinical medicine. The methodology comes with several helpful advantages. First, unlike several other proposed techniques [5,6,7], it is agnostic to the specific machine learning techniques employed to develop the predictive models. Second, it makes no assumptions about the underlying distribution of data. Third, it is available as an open-source tool. Fourth, it is flexible enough to manage single sample data (as in the case of COVA and BAI) or time series data (as in the case of ST). Finally, it provides precision and accuracy guarantees within pre-specified confidence ranges.

Each of the three datasets used highlights different features of SSAML (Table 1). For BAI and COVA, one sample was obtained per patient. For ST, there were multiple samples per patient (i.e., repeated measures). BAI was analyzed using survival statistics while COVA and ST were not. The number of events was computed for COVA, whereas for ST and BAI the number of patients was employed. Each of these three datasets were derived from different types of ML models. Finally, as seen in Figure 2, each of these models had different degrees of calibration and precision compared with the ground truth. In short, many different situations are well handled by SSAML.

The limitations of SSAML should be noted. Depending on the dataset, this method can be computationally expensive, and access to high performance computing may be beneficial. Moreover, if the sample of data is inadequate, or alternatively if one is unable to generate reasonable simulated data, SSAML cannot be expected to be accurate. If insufficient samples are included, or a large number of sample but not enough to fully capture the population distribution, both cases are expected to fail. An overly simplified example illustrates this point. Suppose an ML technique predicts migraine risk from eye movements. Using 1000 sighted participants as the input dataset for SSAML, the number of participants required for validation will be inaccurate if the validation study includes blind participants. In other words, the variability of the output of any model needs to be captured with a representative sample of data so that SSAML can predict a reasonable sample size. As with any statistical model, the results of SSAML depend on the quality and representativeness of the data being used. In addition, it is important to emphasize that SSAML is not designed to predict the sample size for traditional trials designed around hypothesis testing (such as traditional drug vs. placebo trials). Rather, SSAML is focused on determining the sample size needed to validate a machine learning predictive model (such as a disease diagnosis prediction or time-to-death model).

## 5. Conclusions

Whenever a machine learning method is found to have potential clinical value, a validation study is required to demonstrate the generalizability of the tool. Due to the complexity of modern machine learning tools, traditional sample size estimators are inadequate for planning validation studies. SSAML supplies a flexible framework for determining an appropriate sample size. Because it is model agnostic, distribution agnostic, and open-source, SSAML can provide a robust estimate of sample size for such validation studies regardless of the details in many contexts. 

## Figures and Tables

**Table 1:** Characteristics of the three example datasets used. BAI = brain age index. COVA = COVID-19 risk study. ST = Seizure Tracker™. In all three datasets, the number of events and number of patients are listed, but only in the COVA dataset we employed an event-based analysis, whereas in BAI and ST we used a patient-based analysis.

Dataset	Machine Learning	# of Patients/# of Events	Disease Model	Outcome	Repeated Measures	Survival Analysis	Event-Based Analysis
BAI [8,10]	Transformed linear regression	4070 patients 3359 events	Aging	Estimate of brain age (used to forecast life expectancy)	N	Y	N
COVA [11]	Ordinal regression	2205 patients 1479 events	COVID-19	Risk of hospitalization, critical illness, or death	N	N	Y
ST [12]	Deep learning	1613 patients 98,119 events	Epilepsy	Risk of seizure within 24 h	Y	N	N

**Table 2:** SSAML result tables from each example dataset (BAI, COVA, ST). BAI = Brain Age Index (BAI) [8,10], COVA = COVID-19 risk Assessment [11], ST = Seizure Tracker™ [12]. Highlighted in bold are numbers that satisfy the requirements: *RWD* < 0.5, |*BIAS*| < 0.05, and *COVP* > 0.95. The number of patients/events that satisfy the requirements for all categories are also highlighted in bold. Conf. int. = confidence interval, *Slope* = calibration slope, *AUC* = area under the receiver operator curve, *C-index* = Harrell’s c-index, *CIL* = calibration-in-the-large, *RWD* = relative width of confidence interval, *BIAS* = bias in estimate compared with “true” value, *COVP* = probability of confidence interval covering “true” value. Note: for the confidence interval used for ST with 20 patients, the actual value was 0.9999; however, due to rounding for three significant digits it is listed as 1.000.

BAI		Number of Participants			
METRIC		500	1000	**1500**	**2000**
Conf. int.		0.997	0.997	0.955	0.955
*RWD*	*slope*	1.602	0.848	**0.444**	**0.382**
*RWD*	*C-index*	**0.157**	**0.110**	**0.061**	**0.052**
*RWD*	*CIL*	**0.196**	**0.134**	**0.073**	**0.063**
*BIAS*	*slope*	−0.053	**−0.024**	**−0.008**	**−0.008**
*BIAS*	*C-index*	**−0.001**	**−0.001**	**−0.002**	**−0.001**
*BIAS*	*CIL*	**0.005**	**0.002**	**0.002**	**0.001**
*COVP*	*slope*	**0.981**	**0.989**	**0.953**	**0.958**
*COVP*	*C-index*	**0.998**	**0.993**	**0.955**	**0.958**
*COVP*	*CIL*	**0.994**	**0.994**	**0.959**	**0.951**
**COVA**		Number of events			
METRIC		125	**150**	**175**	**200**
Conf.int.		0.955	0.955	0.955	0.955
*RWD*	*slope*	0.557	**0.486**	**0.421**	**0.378**
*RWD*	*AUC*	**0.149**	**0.132**	**0.116**	**0.104**
*RWD*	*CIL*	**0.285**	**0.248**	**0.218**	**0.197**
*BIAS*	*slope*	**−0.005**	**0.000**	**0.000**	**0.002**
*BIAS*	*AUC*	**−0.001**	**−0.001**	**0.000**	**0.001**
*BIAS*	*CIL*	**−0.011**	**−0.005**	**−0.004**	**−0.007**
*COVP*	*slope*	**0.966**	**0.964**	**0.956**	**0.973**
*COVP*	*AUC*	**0.968**	**0.979**	**0.972**	**0.974**
*COVP*	*CIL*	**0.977**	**0.971**	**0.966**	**0.977**
**ST**		Number of patients			
METRIC		20	**40**	**60**	**80**
Conf.int.		1.000	0.997	0.997	0.997
*RWD*	*slope*	1.001	**0.324**	**0.187**	**0.131**
*RWD*	*AUC*	**0.378**	**0.205**	**0.168**	**0.146**
*RWD*	*CIL*	**0.370**	**0.165**	**0.129**	**0.107**
*BIAS*	*slope*	0.076	**0.022**	**0.010**	**0.007**
*BIAS*	*AUC*	**0.046**	**0.023**	**0.015**	**0.011**
*BIAS*	*CIL*	**−0.026**	**−0.010**	**−0.008**	**−0.005**
*COVP*	*slope*	**0.990**	**0.996**	**0.996**	**0.991**
*COVP*	*AUC*	**0.977**	**0.965**	**0.972**	**0.981**
*COVP*	*CIL*	**0.997**	**0.992**	**0.996**	**0.997**

## Data Availability

The original datasets (ST, COVA and BAI) are not publicly available due to privacy concerns. Collaboration is an option for reasonable projects. Open-source code for SSAML and all the simulations are available (https://github.com/GoldenholzLab/SSAML).
